# Why do seismic hazard models worldwide appear to overpredict historical intensity observations?

**DOI:** 10.1126/sciadv.adj9291

**Published:** 2024-05-03

**Authors:** Leah Salditch, Molly Margaret Gallahue, Seth Stein, James Scott Neely, Norman Abrahamson, Susan Elizabeth Hough

**Affiliations:** ^1^ U.S. Geological Survey, Golden, CO, USA.; ^2^ Department of Earth and Planetary Sciences, Northwestern University, Evanston, IL, USA.; ^3^ Institute for Policy Research, Northwestern University, Evanston, IL, USA.; ^4^ Department of Civil and Environmental Engineering, University of California, Berkeley, Berkeley, CA, USA.; ^5^ U.S. Geological Survey, Pasadena, CA, USA.

## Abstract

Probabilistic seismic hazard assessments (PSHAs) provide the scientific basis for building codes to reduce damage from earthquakes. Despite their substantial impact, little is known about how well PSHA predicts actual shaking. Recent PSHA for California, Japan, Italy, Nepal, and France appear to consistently overpredict historically observed earthquake shaking intensities. Numerical simulations show that observed shaking is equally likely to be above or below predictions. This result from independently developed models and datasets in different countries and tectonic settings indicates possible systematic bias in the hazard models, the observations, or both. Analysis of possible causes shows that much of the discrepancy is due to a subtle and rarely considered issue: the conversion equations used in comparing the models—which forecast shaking as peak ground acceleration or velocity—and observations—parameterizations of qualitative shaking reports. Historical shaking reports fill a crucial data gap, but more research is warranted on how qualitative observations relate to instrumental shaking measures for earthquakes.

## Teaser

Seismic hazard models predict higher shaking intensities than observed due to biased conversion equations used to compare them.

## INTRODUCTION

Modern earthquake science strives to understand earthquake occurrence (*1*) and the resulting ground motions that earthquakes produce. Best-available science is used to develop probabilistic seismic hazard assessment (PSHA) models that forecast the level of ground motions expected to be exceeded with a certain probability in a given time span, e.g., 10 or 2% probability of exceedance in 50 years (*2*, *3*). PSHA models are a societally impactful product of earthquake science, establishing the basis for building codes, insurance rates, and mitigation priorities.

**Fig. 1.**
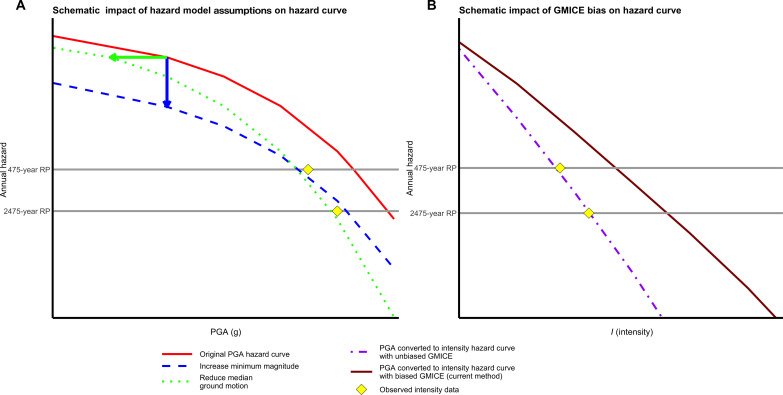
Schematic probabilistic seismic hazard assessment (PSHA) hazard curves compared to observations. Curves to the right of the yellow diamonds (observations) predict higher shaking than observed in the historical catalog. Ideally, curves pass through the diamonds. (**A**) Alternative hazard model assumptions—reducing the median ground motion predicted by ground-motion models or increasing the minimum magnitude of earthquakes included in the hazard calculation—could make the predicted hazard more consistent with the observed shaking data at this site. Arrows show how adjustments change the original hazard curve; 475-year and 2475-year return periods (RP) are shown. PGA (g) is peak ground acceleration measured in units of g (m/s^2^). Annual hazard, also known as annual frequency of exceedance, is equal to 1/RP. (**B**) Changing from a biased GMICE to an unbiased GMICE would reduce the predicted hazard. After (*29*).

PSHA forecasts involve substantial uncertainties due to limitations in knowledge of how the earth has behaved and will behave. Assumptions regarding the earthquake sources (the location, rate, and magnitude of future earthquakes) and ground-motion models (the characteristics of ground motion at a given site resulting from an earthquake) yield substantial uncertainties in the predicted hazard. These effects are illustrated by sample curves showing the expected hazard (shaking) at a site over different time periods (return periods) (Fig. 1A) under different assumptions, one of which is chosen as “the” hazard at the site.

Hazard models show the resulting predicted hazard at many sites over a large area. To understand how changes in inputs affect PSHA, consider a hazard curve for a single site. Figure 1A illustrates how changes in PSHA inputs will change the hazard curve. If modeled median ground motions predicted by ground-motion models are lowered, hazard curves shift horizontally, lowering the expected hazard. If the minimum magnitude of earthquakes included in the hazard calculations increased, hazard curves shift vertically, also lowering the expected hazard.

Such uncertainties have fueled debate and concerns about the PSHA approach (*4*–*6*). Alternatively, deterministic seismic hazard assessment that considers expected shaking from specific future earthquakes is sometimes used (*7*). Nonetheless, PSHA is primarily used worldwide for insurance and building codes, including for critical infrastructure like nuclear waste repositories (*8*, *9*).

Despite their importance, little is known about how well hazard models and the corresponding maps predict future shaking. Because large earthquakes are infrequent in any area, prospective assessment of PSHA models using earthquakes after a model was made requires impractically long time frames. Hence, a useful approach to evaluate models involves retrospective or hindcasting assessments, comparing PSHA to compilations of historically observed shaking that were available when the models were made but typically not used directly in making hazard models (*10*–*13*).

Hindcasting seismic hazard with long historical datasets relies on seismic intensity, a qualitative measure of ground shaking directly related to damage, which can be inferred from historical records and so gives data before the advent of instrumental seismology in about 1900 (*14*, *15*). Historical observations are individually assigned an intensity value by one or more experts and, despite their inherently subjective nature, are very useful (*16*). Intensity scales vary by region, with Modified Mercalli Intensity favored in the United States, while the Mercalli-Cancani-Seiberg (MCS) and European Macroseismic Scale are favored in Europe. Japan has its own intensity scale, JMA, named after the Japan Meteorological Agency. Apart from JMA, most modern intensity scales are similar, with a common genesis (*17*). Intensity is also used in modern information products communicating hazard to the public, including earthquake early warning (*18*), Prompt Assessment of Global Earthquakes for Response loss impact estimates (*19*), and rapid ShakeMap in the United States (*20*).

PSHA models predict accelerations or velocities of ground motions that would be recorded instrumentally and are relevant to engineering concerns. To compare the PSHA results with intensity observations, predicted ground motions [measures of shaking such as peak ground acceleration (PGA) or peak ground velocity (PGV)] are converted to intensity using a ground-motion to intensity conversion equation (GMICE) (Fig. 1B) (*21*). The U.S. Geological Survey’s ShakeMap system uses GMICEs because intensity information helps constrain ShakeMaps (*20*).

A seismic hazard map is a representation of the hazard model for a given return period, i.e., a spatial representation of individual hazard curves sampled at the same point on the *y* axis (Fig. 1). One can compare PSHA map predictions with observations via the fractional exceedances (the fraction of sites considered at which the highest ground shaking in a given time interval exceeded the modeled value). At a point on the hazard map, the probability *p* that during *t* years of observations shaking will exceed (at least once) a value expected in a *T*-year return period is assumed to be described by a Poisson distribution, *p* = *1* − exp(−*t*/*T*) (*3*). Under the Poisson assumption typically used in PSHA, the fraction of sites within a map at which observed shaking exceeds the mapped value is expected to behave the same way (*22*). Assuming that the observations at the different sites are statistically independent, the shaking shown on a map with a *T*-year return period would be exceeded at 10% of the sites in *t* = *T*/10 years, 39% in *t* = *T*/2 years, and 63% in *t* = *T* years.

## RESULTS

**Fig. 2.**
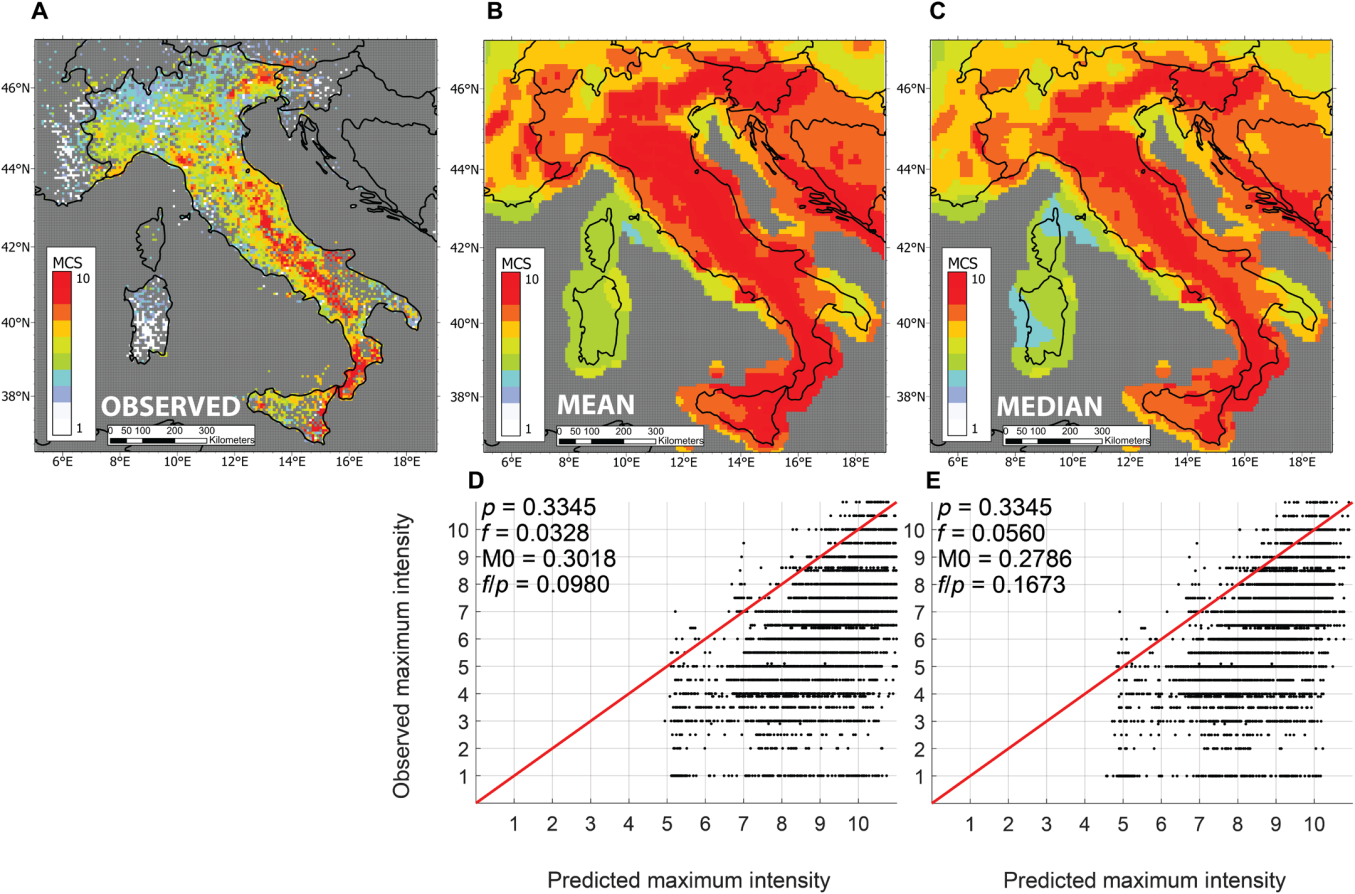
Comparison of shaking data and hazard models for Italy. (**A**) A map of observed intensities in the MCS intensity scale from the DBMI15v2 database (Data and materials availability), spanning 1008 years. (**B** and **C**) Mean and median values of the ESHM20 hazard model for 2% in 50 years (2500-year return period), converted from PGA to MCS intensity using GMICEs. (**D** and **E**) Results of the comparison with accompanying metrics. Although the metrics improve somewhat using the median hazard, a major misfit remains.

**Fig. 3.**
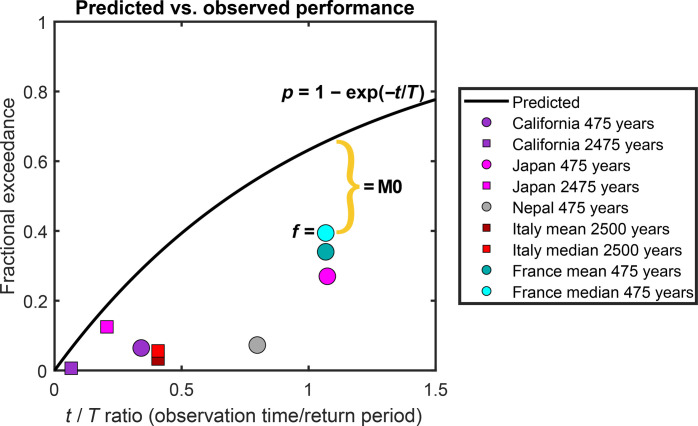
PSHA model performance. Predictions follow *p* = 1 − exp(−*t/T*), where *t* is the observation duration and *T* is the return period of the map. Symbols represent the observed fractional exceedances, *f*, color coded by region. The M0 metric can be visualized as the distance between *f* and *p* (solid black line) for a given *t/T* (Table 1). Numbers following regions in the legend are map return periods (circles have 475-year return periods, and squares have 2475-year or 2500-year return periods). All hazard models are for mean hazard, unless otherwise noted (median hazard is assessed for France and Italy). Using median hazard slightly improves metrics in both cases here. Notably, all cases here fall below the predicted line, indicating that observed shaking is less than predicted by the hazard models.

In recent years, a growing number of studies have compared PSHA models to compilations of historically observed shaking intensities (Table 1). The hazard models and intensity datasets have been developed separately, generally by national agencies. For example, Fig. 2 compares a 1008-year long record of shaking data for Italy to the predictions of the European Seismic Hazard Model 2020 (ESHM20) for a 2500-year return period (Data and materials availability). Both visual inspection and the metrics show that the models of Italy substantially overpredict the observed shaking. Models for California, Japan, Italy, Nepal, and France also appear to consistently overpredict historically observed intensities, as shown by the data values below the predicted curve (Fig. 3). The M0 metric is visualized as the distance between an observed fractional exceedance, *f*, and the predicted exceedance, *p*, for a given ratio of observation duration to map return period. In addition to the propensity for model overprediction, no consistent improvement appears with longer ratios of observation duration to map return period. These results are contrary to numerical simulations showing that if a model’s assumptions about earthquake occurrence and ensuing ground motion are approximately correct, the observed shaking is equally likely to be above or below predictions and that observations should be closer to the prediction as observation duration (*t/T*) increases, and the largest earthquakes and shaking are increasingly likely to have occurred (*23*).

**Table 1.** Hazard models and their performance metrics from comparison with seismic intensity observations.

Model return period *T* (years)	Model prob. of exceedance	Region (reference)	*p*	*f*	*f*/*p* ratio (ideally 1)	M0 (ideally 0)	No. of points	No. of exceedances	Length of obs. *t* (years)	*t/T* ratio
475	10% in 50 years	California (*13*)	0.2892	0.0644	0.2227	0.2248	2687	173	162	0.3411
475		Japan (*12*)	0.6600	0.2700	0.4091	0.3900	n/a	n/a	510	1.0737
475		Nepal (*32*)	0.5501	0.0728	0.1323	0.4772	961	70	379	0.7979
475		France—median (*32*)	0.6561	0.3941	0.6007	0.2623	3995	1575	507	1.0674
475		France—mean (*32*)	0.6561	0.3402	0.5183	0.3163	3995	1359	507	1.0674
2475	2% in 50 years	California (*13*)	0.0634	0.0063	0.0994	0.0570	2687	17	162	0.0655
2475		Japan (*12*)	0.1900	0.1250	0.6579	0.0300	n/a	n/a	510	0.2061
2475		Italy (*11*)	0.5859	0.0025	0.0043	0.5864	800	2	2200	0.8889
2500		Italy—median (this study)	0.3345	0.0560	0.1674	0.2786	5307	297	1008	0.4072
2500		Italy—mean (this study)	0.3345	0.0328	0.0980	0.3018	5307	174	1008	0.4072

These results indicating that PSHA systematically overpredicts observed shaking prompted research into the cause(s) of the discrepancy. Because the discrepancy arises in different countries and tectonic environments, it is likely not caused by inaccurate seismic source or ground-motion models, which vary between areas. Instead, it is likely due to an effect that is common to different areas.

## DISCUSSION

We investigated this question primarily using a dataset from the California Historical Intensity Mapping Project (CHIMP) (*13*) that we compiled, giving us full information about the data and thus the ability to conduct detailed analyses. First, we considered the influence of local geology on shaking and hazard via the commonly used proxy Vs30 (the time-averaged shear-wave velocity in the upper 30 m of the crust) and found that these site effects were not a substantial contributor to the observed discrepancy, as including them tended to increase the discrepancy by up to approximately 10% rather than decrease it (*24*). Next, we explored issues arising from different magnitudes of completeness between the data. CHIMP catalog completeness may be as high as ~magnitude (M) 6.6 but is no lower than M 6, whereas the hazard model [U.S. Geological Survey National Seismic Hazard Model 2018 (*25*)] has minimum magnitude ~M 5. Numerical experiments were done by recalculating the hazard model with minimum magnitude M 6 and comparing it to a modified CHIMP catalog consisting only of observations from M 6+ (*26*). Correcting for the different magnitudes of completeness reduces the discrepancy by approximately 10 to 15%, which is not enough to bring the models and data in alignment. Similar results were obtained when recalculating the model with a minimum magnitude of *M* 6.6. Thus, completeness inconsistencies and site effects contribute to, but are not the primary cause of, the discrepancy between predicted and historically observed shaking in California.

Another contributor to the discrepancy might be the choice of hazard fractile. PSHA studies use a suite of alternative models based on various assumptions for the earthquake process and wave propagation. Because the distribution of hazard results from the alternative models is skewed to the high side, the mean hazard is larger than the median. To penalize uncertainty, hazard models are typically given as the mean of the total hazard estimated for the alternative models, although the median is likely more representative of the best available science and should be used to assess overall model performance, as it is usually less biased by outliers (*27*). To further account for potential biases, consideration should be given to the full hazard distribution including epistemic uncertainty whenever possible (*28*–*30*).

An example of the difference between using the mean or the median hazard fractiles is illustrated for Italy (Fig. 2). For the comparison, we used the ESHM20 mean and median hazard estimates of PGA for a 2500-year return period, which we converted from PGA to MCS intensity using GMICEs (*31*). Although using the median hazard instead of the mean improves the fit (M0 is reduced by 10%), a substantial misfit remains. Similarly, Gallahue (*32*) found that using the median hazard for California reduced the discrepancy between the models and data by only about 10%, and Salditch (*33*) found that median hazard models for France reduced the discrepancy by 14%.

Thus, a number of possible effects are not the major contributors to the discrepancy. Instead, the most substantial contribution arises from a subtle and unexpected source, the GMICE used to convert ground motions (e.g., PGA and PGV) in the hazard models to seismic intensities.

Current GMICEs have been developed using standard methodology for the past 80 years, with updates mainly reflecting larger datasets (*34*). The GMICEs perform quite well on average for individual earthquakes. A recent study (*35*) finds that the GMICEs work well for estimating intensity from median ground motions, but the intensities estimated by the GMICEs can have large bias for ground-motion values away from the medians (Fig. 1B). Because the main contribution to the PSHA results used for building codes is typically from above-average ground motions, current GMICEs lead to overestimation of the expected intensity. Correcting for this bias in the GMICE in California substantially reduces the discrepancy, indicated by a decrease of the M0 metric of 40 to 70% (*32*). Because a similar bias will occur in GMICEs developed for different regions using the same standard methodology, improved conversion equations will likely reduce the discrepancy between hazard models and data elsewhere.

Other, presumably lesser, effects may also play some role. Intensity values themselves always merit careful attention: In any area, historically observed intensities may be biased low by incompleteness of compiled intensities. Intensities can also be biased high due to various factors (*15*). However, the different regional datasets were compiled separately by different researchers using different techniques. A growing number of recent studies also indicate that hazard models may be biased high by the use of regionally averaged ground-motion models rather than path-dependent models that capture differences in ground motions due to the three-dimensional Earth structure (*36*). Development of path-dependent models will tend to reduce probabilistic hazard (*37*) and is an active area of research (*38*).

However, the results of the work of Salditch (*33*) and this study suggest that neither PSHA models nor historically observed intensities are systematically biased. Instead, apparent inconsistencies are mostly due to the conversion equations used to compare them. These results underscore the potential pitfall of equating instrumental shaking metrics like PGA or PGV with hazard or damage potential (*39*). The effect of shaking on people and the built environment depends not only on PGA or PGV but also on other factors such as shaking duration, frequency content, and structural building conditions. Further improvements in GMICEs, as outlined by Gallahue and Abrahamson (*35*), will be important both to better evaluate current PSHAs and to better characterize the damage potential of shaking caused by future earthquakes.

From the standpoint of mitigating earthquake risk, it is encouraging that much of the apparent overprediction of earthquake hazards results from the conversion equations used in comparing the models and observations rather than a systematic effect in the earthquake hazard modeling approach. Thus, although any given hazard model may overpredict or underpredict shaking due to chance or inaccurate source or ground-motion parameters (*4*, *23*), we find no evidence for underlying systematic problems with the hazard modeling.

## MATERIALS AND METHODS

For a typical hazard model, *t* is set to 50 years (a standard interval of interest), and maps are produced for a specific return period, corresponding to a certain probability of exceedance, e.g., a 475-year return period corresponds to a 10% probability of exceedance. When evaluating hazard map performance, we use the map’s return period (the defining characteristic of the map) and the duration *t* of the observed dataset, which adjusts *p*. For example, in Italy, comparing a 2500-year hazard map to the observed data that span 1008 years, the Poisson equation gives: *p* = *1* − exp(−1008/2500) = 0.33 (Data and materials availability). Hence, for the observed data spanning 1008 years, we expect that 33% of sites will exceed the ground motion shown on the 2500-year map, rather than the original 2% expected in 50 years (Fig. 2).

We quantify the comparison between hazard models and observations using a metric M0 = |*p* − *f*|, the absolute value of the difference between the predicted fractional exceedance *p* and the observed exceedance *f* (*11*). M0 is ideally 0, indicating a model that perfectly predicts the observed fraction of exceedances. A metric using the ratio of observed to predicted exceedances is also informative (*40*). *f*/*p* = 1 is ideal, *f*/*p* > 1 means the model underpredicted observed shaking, and *f*/*p* < 1 means the model overpredicted observed shaking. Acceptable variations from the hazard model target of around 20% have been suggested (*22*).
